# *HOXA9* is a novel myopia risk gene

**DOI:** 10.1186/s12886-019-1038-9

**Published:** 2019-01-23

**Authors:** Chung-Ling Liang, Po-Yuan Hsu, Cheryl S. Ngo, Wei Jie Seow, Neerja Karnani, Hong Pan, Seang-Mei Saw, Suh-Hang H. Juo

**Affiliations:** 1 Department of Ophthalmology, Asia University Hospital, Taichung, Taiwan; 2 Department of Optometry, College of Medical and Health Science, Asia University, Taichung, Taiwan; 3 Center for Myopia and Eye Disease, Department of Medical Research, China Medical University Hospital, Taichung, Taiwan; 4 Bright-Eyes Clinic, Kaohsiung, Taiwan; 5 Department of Ophthalmology, National University Hospital, Singapore, Singapore; 6 Saw Swee Hock School of Public Health, National University of Singapore and National University Health System, Singapore, Singapore; 7 Singapore Institute for Clinical Sciences (SICS), A*STAR, Brenner Centre for Molecular Medicine, Singapore, Singapore; 8 Singapore Eye Research Institute, Singapore, Singapore; 9 The Ophthalmology & Visual Sciences Academic Clinical Program, DUKE-NUS Graduate Medical School, Singapore, Singapore; 10 Graduate Institute of Biomedical Sciences, Singapore, Singapore; 11 Institute of New Drug Development, Singapore, Singapore; 12 Drug Development Center, China Medical University, Taichung, Taiwan

**Keywords:** HOXA9, PAX6, microRNA-328, Myopia

## Abstract

**Purpose:**

A recent meta-analysis revealed *PAX6* as a risk gene for myopia. There is a link between *PAX6* and *HOXA9*. Furthermore, *HOXA9* has been reported to activate TGF-β that is a risk factor for myopia. We speculate *HOXA9* may participate in myopia development.

**Methods:**

The Singapore GUSTO birth cohort provides data on children’s cycloplegic refraction measured at age of 3 years and their methylation profile based on the umbilical cord DNA. The *HOXA9* expression levels were measured in the eyes of mono-ocular form deprivation myopia in mice. The plasmid with the mouse *HOXA9* cDNA was constructed and then transfected to mouse primary retinal pigment epithelial (RPE) cells. The expression levels of myopia-related genes and cell proliferation were measured in the *HOXA9*-overexpressed RPE cells.

**Results:**

A total of 519 children had data on methylation profile and cycloplegic refraction. The mean spherical equivalent refraction (SE) was 0.90D. Among 8 SE outliers (worse than -2D), 7 children had *HOXA9* hypomethylation. The *HOXA9* levels in the retina of myopic eyes was 2.65-fold (*p* = 0.029; paired t-test) higher than the uncovered fellow eyes. When *HOXA9* was over-expressed in the RPE cells, *TGF-β*, *MMP2*, *FGF2* and *IGF1R* expression levels were dose-dependently increased by *HOXA9*. However, over-expression of *HOXA9* had no significant influence on *IGF1* or *HGF* expression. In addition, *HOXA9* also increased RPE proliferation.

**Conclusion:**

Based on the human, animal and cellular data, the transcription factor *HOXA9* may promote the expression of pro-myopia genes and RPE proliferation, which eventually contribute to myopia development.

## Introduction

Myopia is a common eye condition worldwide, and its prevalence varies widely among populations and ages [1–3]. Both genetic and environmental factors contribute to the development of myopia [4]. Several myopia susceptibility genes have been reported based on genetic association studies as well as gene expression studies [5–7]. Recently, data from meta-analysis of genome-wide association studies further reported newly identified genetic loci [8–10]. On the other hand, several environmental risk factors were reported to be associated with myopia. Outdoor activity is demonstrated to be a protective factor against myopia onset and progression in school children [11–13]. Furthermore, meta-analysis also provides evidence to support that the interaction between genetic and environmental factors contributes to myopia development [14].

We previously reported that microRNA-328 (miR-328) can bind to *PAX6* mRNA and negatively regulate *PAX6* expression, which leads to myopia development [2, 15]. A recent meta-analysis revealed *PAX6* as a myopia risk gene [16]. *MEIS1* is a transcription factor that regulates the retina [17–19] and lens development [20, 21]. It has been shown that *PAX6* and *MEIS1* can simultaneously regulate eye development [20]. On the other hand, *MEIS1* can form a complex with *HOXA9* in myeloid cells [22]. *HOXA9* can transcriptionally activate transforming growth factor-β (TGF-β) [23] and TGF-β signaling has long been implied as a risk factor for myopia [24]. All these three genes (*PAX6*, *MEIS1* and *HOXA9*) are homeobox genes. In addition, our methylation profiles of genomic DNA showed aberrant methylation at *HOXA9* in myopia of preschool children (see details in the Result section). Although there has been no report regarding the role of *HOXA9* gene in any eye diseases or myopia, we speculate *HOXA9* may participate in myopia development because of the aforementioned findings. In the present study, we investigated the effects of *HOXA9* on myopia using human, animal and cellular samples.

## Method

### Human studies

#### DNA methylation study

We used the data collected from the GUSTO birth cohort, which was previously described [25]. Children with any known or determined eye conditions including strabismus, eye infection, eye injury, facial nerve palsy, developmental anomaly and other such eye related conditions were not included. The National Health Group’s Domain Specific Review Board and the SingHealth Centralized Institutional Review Board approved this study. The parents or legal guardians gave informed written consent. The present study was conducted according to the tenets of the Declaration of Helsinki.

Genomic DNA was extracted from infant umbilical cords of GUSTO samples collected at birth and was profiled using the Infinium Human Methylation450 BeadChip arrays. The details of methylation profiling can be found in our previous publication [26]. Data were processed by signal correction and adjustment for different color channels as described by Pan et al. [27]. A total of 160,418 CpGs of the 519 subjects were finally used for further analysis [26–29].

#### Eye measurements performed at 3 years old

Out of 1236 recruited participants, 925 children (74.8%) attended the third year clinic visit. Axial length (AXL) was measured in 764 children (61.5%), and a cycloplegic refraction was performed in 574 children (46.3%). Cycloplegic autorefraction was performed with a table-mounted autorefractor (Model RK-F1; Canon, Tokyo, Japan). Spherical equivalent refraction (SE) for each eye was calculated as sphere power plus half cylinder power. We used data from right eyes only, due to the high correlation between the right and left eyes (Spearman rho: 0.88 for SE and 0.96 for AXL). Myopia was defined as a SE of at least − 0.5 diopter (D). The analysis was based on 519 children who had data on methylation profile and cycloplegic refraction.

### Animal studies

#### FDM mice and measure of ocular axial length

We used a well-documented method to induce mono-ocular form deprivation myopia (FDM) in mice [30]. The C57BL/6J mice were purchased from the National Laboratory Animal Center, Taiwan. Mice were maintained in a temperature-controlled (25 °C) facility with a strict 12 h: 12 h light: dark cycle. The right eyes of mice were covered from age of 23 days to 51 days (i.e. covered for 4 weeks) to induce myopia, while the left eyes were uncovered. All the animals were euthanized on day 51 mice by using an overdose of isoflurane anesthesia, and both eyes were dissected for AXL measurement. To euthanize the mice, animals were placed into clear, plastic cages. When the mice were added to the cage, isoflurane (Attane™, Panion & BF Biotech Inc., Taiwan) was applied to the absorbent paper towels that were in the cage, and the cage was immediately sealed. The operator continuously monitored the mice for their respiration, color, and movement. If a mouse showed loss of consciousness, respiratory arrest and no heart beat by direct cardiac palpation, we confirmed that a mouse had died.

In order to reduce human errors and bias while measuring AXL, we developed a software to automatically calculate the AXL of dissected eyes. First, a pair of dissected eyes was placed on a slide for photo picture under a dissecting microscope. Then the K-means clustering algorithm was employed to separate the eyes from the background matrix to obtain a cleaned image. Finally, the software split the two eyes into two contours, and the ellipse fitting algorithm is used to fit on both contours. The contour’s longest vertical line is defined as the AXL.

The animal care guidelines are comparable with those published by the Institute for Laboratory Animal Research (NIH Publications No 8023, revised 1978). The animal research in this study was approved by the Animal Care and Ethics Committee at China Medical University, Taiwan.

### Cellular studies

#### Human retinal pigment epithelial cell culture

The human retinal pigment epithelial (ARPE-19) cell line was obtained from Bioresource Collection and Research Center (BCRC) (Hsinchu, Taiwan) which derived from American Type Culture Collection (ATCC, Manassas, VA; ATCC number: CRL-2302). ARPE-19 cells were cultured in DMEM/F12 medium (Gibco-BRL, Gaithersburg, MD) supplemented with 10% FBS (Gibco-BRL), 50 units/mL penicillin, and 50 mg/mL streptomycin. The cells were seeded on a 12-well plate (10^5^ cells/well) and were transfected with miR-328 by HiPerfect transfection reagent (Qiagen, Valencia, California, USA). Four hours after transfection, cells were changed into normal culture medium.

#### Mouse primary RPE cell

Eyes were dissected from male C57BL/6J mice aged 6–10 weeks. The dissected eye was incised from the optic nerve insertion site to remove lens, retina and cornea without disrupting the underlying retinal pigmented epithelium. The left behind RPE/choroid-sclera complex was placed in digestion buffer 0.25% trypsin in DMEM for 1 h at 37 °C with gentle digestion. FBS was added to the tissue to terminate the digestion, and then the tissue was washed twice with 1x PBS and subject to centrifuge (1500 rpm, 5 min at 4 °C). After removal of the supernatant, culture medium was added to the tube and RPE cells were cultured in incubator at 37 °C. RPE culture medium contained N2 supplement (Gibco) 1:100 mL/mL, penicillin–streptomycin (Sigma-Aldrich) 1:100 mL/mL, nonessential amino acid solution (Sigma-Aldrich) 1:100 mL/mL, hydrocortisone (20 μg/L, Sigma-Aldrich), taurine (250 mg/L, Sigma-Aldrich), and triiodo-thyronin (0.013 μg/L, Sigma-Aldrich) with 5% FBS [31]. To obtain single cells, the cells were grown on a gelatin pre-coated 24-well plate for 2 h.

#### Immunocytochemistry for mouse RPE cells

Immunostaining was used to confirm that the isolated cells were the mouse RPE. For immunostaining, cells were fixed in 4% paraformaldehyde for 30 min and washed three times with cold PBS. The cells were permeabilized for 30 min with 0.1% Triton X-100-BSA. The cells were washed with PBS and blocked with BSA for 30 min at room temperature. Immunofluorescence staining was performed using anti-RPE-65 (1:200, Abcam, Cambridge, UK; catalog ab78036) [32] and anti-ZO-1 (1:200, Abcam; catalog ab59720) [33] antibodies according to manufacturer’s guide. Cells were counterstained with 40, 6-diamidino-2-phenylindole (DAPI) to identify the nuclei.

#### miRNA transfection

The cells (ARPE-19 and mouse RPE) were seeded on a 12-well plate (10^5^ cells/well) and were transfected with different doses of miRNA-328 (1, 5, 10, 25 and 50 nM) or control miRNA by HiPerFect transfection reagent (Qiagen). Four hours after transfection, cells were changed into normal culture medium. After incubation for 24 h, the cells were harvested and the lysates were utilized for protein detection to verify the efficacy of miRNA-328.

#### Construction and transfection of HOXA9 cDNA

*HOXA9* (NP_034586.1) cDNA was cloned into the pIRES2-EGFP vector (BD Biosciences Clontech, Palo Alto, CA, USA) to form the construct of pIRES2-EGFP-*HOXA9* plasmid. The plasmid was transformed into DH5α competent cells and cultured overnight on a Luria broth agar plate containing kanamycin in a 37 °C constant temperature incubator. Single colonies were picked from the plate, and plasmid DNA was extracted according to the manufacturer’s protocol (QIAGEN). The sequences of constructs were confirmed by DNA sequencing. Cells below passage 10 were used in all experiments. To conduct the transfection experiments, mouse RPE cells were seeded on a 12-well plate at a density of 1 × 10^5^ cells/well. After achieving 70% confluence in a well, pIRES2-EGFP or pIRES2-EGFP-HOXA9, was transfected with Lipofectamine 2000 (Invitrogen, Gaithersburg, MD, USA). After incubation for 24 h, the cells were harvested, and the lysates were utilized for western blot to verify efficacy of HOXA9 overexpression in the mouse RPE cells.

#### Cell viability assay

The WST-1 reagent (diluted 1:10 in growth medium) was added for 2 h as described in the instruction manual (Roche, Mannheim, Germany). Viable cell mass was determined by the optical density measurement by a microplate reader at 450 nm, using 600 nm as a reference wavelength.

#### EdU proliferation assay

To assess cell proliferation, the mouse primary RPE cells were seeded on a 12-well plate and then transfected with pIRES2-EGFP-HOXA9 by Lipofectamine 2000 (Invitrogen). Four hours after transfection, cells were changed into normal culture medium. Twenty-four hours after transfection, cell proliferation rate was detected using the incorporation of 5-ethynyl-29-deoxyuridine (EdU) with the Click-iT EdU Microplate Assay Kit (Thermo Scientific, Waltham, MA, USA). EdU incorporated into DNA was coupled to Oregon Green-azide and then detected using an HRP-conjugated anti-Oregon Green antibody and Amplex UltraRed. Fluorescence detected at an excitation/emission wavelength of 490/585 nm was taken as the cell proliferation rate.

#### Real-time PCR, western blot

Total RNA was extracted from ARPE-19 and mouse RPE using the TRIzol® Reagent (Invitrogen). We used 1 μg of starting mRNA (Applied Biosystems, Darmstadt, Germany) and random hexamers to create cDNA. The sequences of PCR primers are shown in Table 1. The relative amount of mRNA of interest was normalized to GAPDH. Real-time PCR was performed on an ABI StepOnePlus™ Real-Time PCR Systems (Applied Biosystems) in duplicate using 5 μl 2× SYBR Green qPCR Master Mix, 0.2 μl primer sets, 1 μl cDNA and 3.6 μl nucleotide-free H2O, which yielded a 10 μl reaction. The protein concentrations were determined by using the Pierce BCA Protein Assay Kit (Thermo Scientific). Primary antibodies against HOXA9 (1:1000, Genetex Inc. CA, USA), TGF-β2 (1:1000, Santa Cruz Biotechnology, Inc. California USA), TGF-β3 (1:1000, Santa Cruz Biotechnology, Inc), MMP2 (1:1000, Genetex Inc), IGF1R (1 μg/ml, Cloud-Clone, TX, USA), FGF2 (1 μg/ml, Cloud-Clone) and α-tubulin (1:5000, ProteinTech Group, Cambridge, UK) were used. The 2ndary antibody was conjugated to the membrane and then incubated with horseradish peroxidase. We used the ECL non-radioactive detection system to detect the antibody-protein complexes by the LAS-3000 imaging system (Fujifilm, Tokyo, Japan). The ImageJ software (NIH) was used for quantitative measure.

#### Statistical analysis

Quantitative data are expressed as the mean ± standard error of the mean. To compare the gene expression levels between the covered eye and uncovered fellow eye of the same mouse, we used paired student t-test. For the cellular studies, differences between multiple groups were analyzed using one-way analysis of variance, followed by Tukey’s post hoc multiple comparisons test. The relative fold change of RNA expression measured by qPCR was calculated by 2^-ΔΔCT^. Statistical analysis was performed using the Prism software, version 5.0 (GraphPad, Inc., La Jolla, CA, USA). A two-sided *P* < 0.05 was considered statistically significant.

## Results

### Human methylation study for pre-school children

The mean of SE in all pre-school children was 0.90D. Since a negative SE is uncommon at this age, we particularly checked 8 children whose SE values appeared to be outliers (more negative than -2D). Among these 8 myopic children, 7 had hypomethylation but one had hypermethylation at the *HOXA9* gene.

### Increased HOXA9 expression in myopic eyes in the FDM animals

**Fig. 1:**
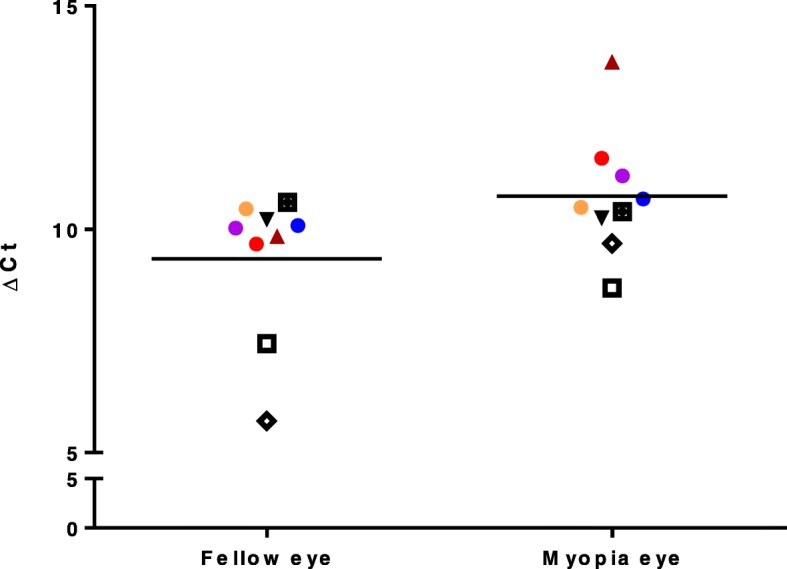
An increase of *HOXA9* expression level in the myopic eyes of FDM mice. *HOXA9* RNA expression level was higher (*p* = 0.029) in the myopic eyes than the fellow normal eyes of FDM mice. The eyes from a same animal have the same color and shape in the figure

We measured *HOXA9* RNA levels in the retina of FDM mice (*n* = 9) by real-time PCR. The expression levels (indicated by delta Ct) in the retina of myopic eyes were significantly higher (*p* = 0.029 by paired t-test) than the uncovered fellow eyes (Fig. 1). The covered myopic eyes had a higher retinal *HOXA9* level by 2.65-fold when compared with the fellow normal eyes. Due to the scarcity of cells in the sclera, we did not have sufficient RNA quantity to measure *HOXA9* expression in the sclera. We also did not collect RPE cells from FDM mice because the sclera and RPE cannot be collected simultaneously from a same eyeball.

**Table 1:** Primers used for quantitative real-time polymerase chain reaction

Gene	Primer
HOXA9 F	5′- CCC CGA CTT CAG TCC TTG C- 3’
HOXA9 R	5′- GAT GCA CGT AGG GGT GGT G- 3’
PAX6 F	5′- TAG CCC AGT ATA AAC GGG AGT G- 3’
PAX6 R	5′- CCA GGT TGC GAA GAA CTC TG- 3’
MMP2 F	5′- CAA GTT CCC CGG CGA TGT C- 3’
MMP9 R	5′- TTC TGG TCA AGG TCA CCT GTC-3’
FGF2 F	5′- GCG ACC CAC ACG TCA AAC TA-3 ‘
FGF2 R	5′- TCC CTT GAT AGA CAC AAC TCC TC-3 ‘
IGF1R F	5′- GAA GAA CGC CGA CCT CTG TTA- 3’
IGF1R R	5′- GCA GCG ATT TGT GGT CCA G ‘
GAPDH F	5′- TGA CCA CAG TCC ATG CCA TC-3 ‘
GAPDH R	5′- GAC GGA CAC ATT GGG GGT AG-3 ‘

### miR-328 promotes HOXA9

**Fig. 2:**
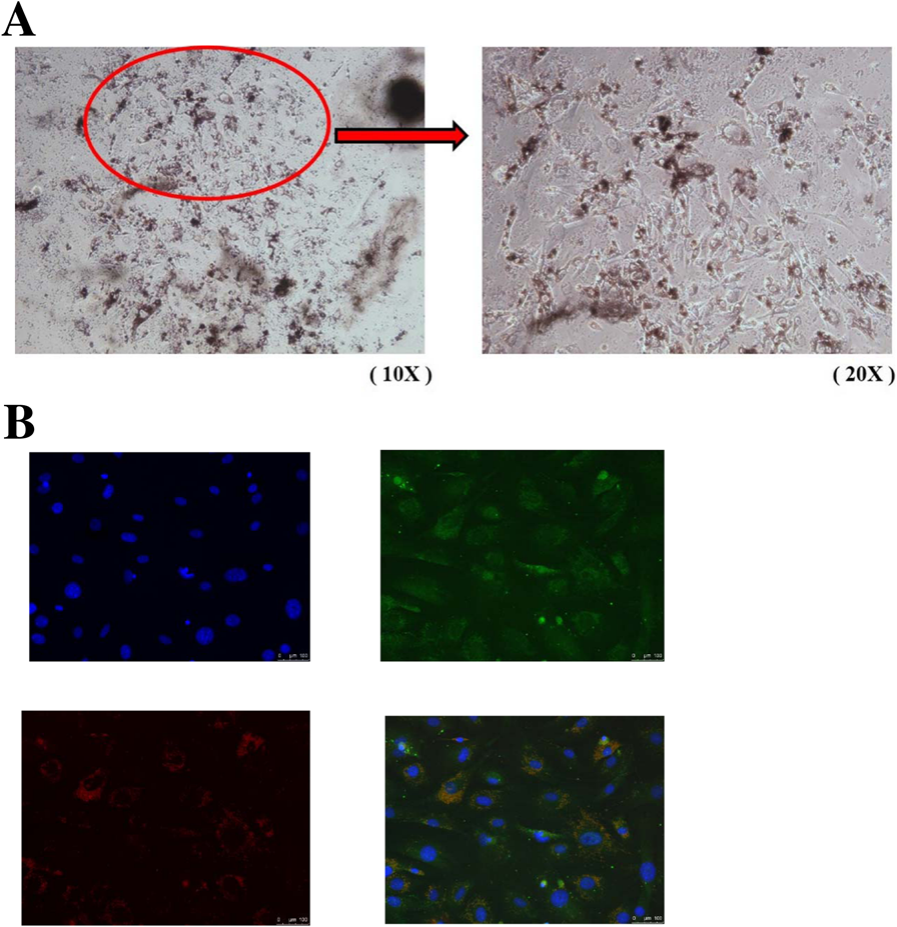
Isolation and culture of mouse primary RPE cells. **a** mouse primary RPE cells (**b**) Immunofluorescent stains confirmed the RPE cells: DAPI (upper left, blue), RPE-65 (upper right, green), ZO-1 (lower left, red) and merge (lower right). Scale bar: 100 μm. 20 x magnification

**Fig. 3:**
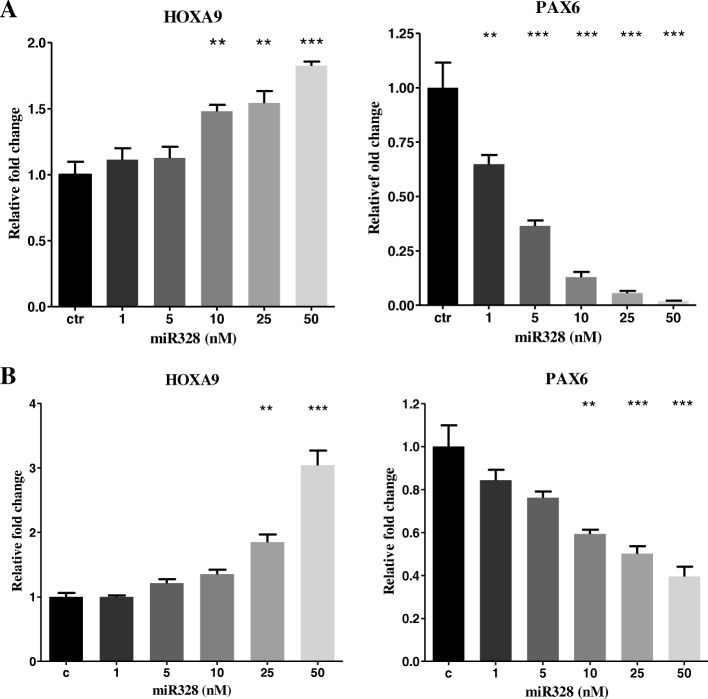
miR-328 affected *HOXA9* and *PAX6* expression in RPE cells. **a** human RPE cells (**b**) mouse primary RPE cells. Transfection of miR-328 to RPE cells increased *HOXA9* but decreased *PAX6* expression levels. *P < 0.05, **P < 0.01 and ***P < 0.001

Although no particular cell model can be used to test myopia development, the RPE cells have been implicated to play a role in myopia [34]. The RPE cells can secrete several growth factors that have been demonstrated to be associated with eyeball elongation [34]. We have shown that over-expression of miR-328 is a risk for myopia [2, 15] and *PAX6* may have a link with *HOXA9*, and therefore we tested whether miR-328 can affect *HOXA9* expression in both human and mouse RPE cells. To confirm that we successfully cultured mouse primary RPE cells (Fig. 2a), RPE-specific markers RPE-65 [32] and ZO-1 [33] were demonstrated in the mouse cells by the immunocytochemistry staining (Fig. 2b). We then showed that miR-328 dose-dependently increased HOXA9 expression in ARPE-19 and mouse primary RPE cells, while miR-328 suppressed PAX6 levels (Fig. 3a and b).

### The effect of over-expression of HOXA9 on RPE cells

**Fig. 4:**
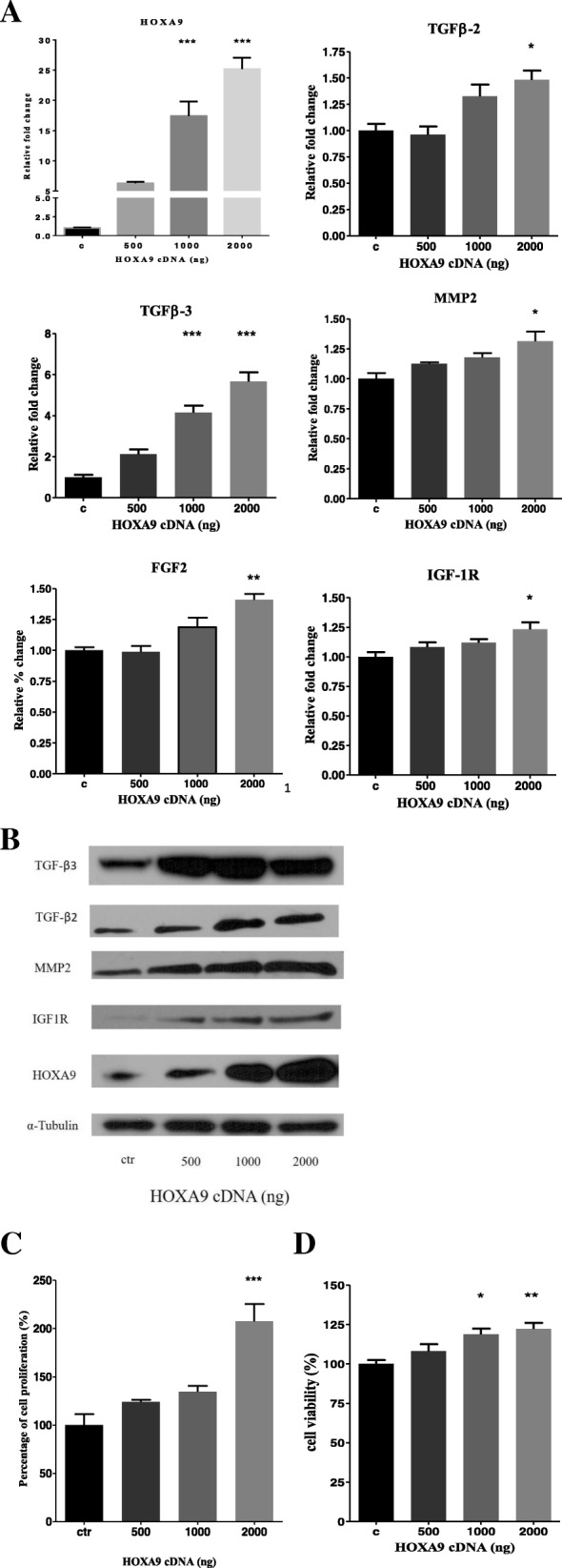
*HOXA9* increased pro-myopia markers and PRE cells. Transfection of HOXA9 plasmid to mouse primary RPE increased (**a** and **b**) RNA and protein levels of *HOXA9, TGF-β2, TGF-β3, MMP2, FGF2* and *IGF1R*, (**c** and **d**) the RPE proliferation and viability in a dose-dependent manner *P < 0.05, **P < 0.01 and ***P < 0.001.

The mouse primary RPE cells were further used to test for the role of over-expressed *HOXA9* in myopia development. Although there is no specific molecular marker for myopia, several molecules have been reported to be associated with myopia, which include TGF-β [24], FGF2 [35], IGF signaling [36, 37], HGF [38, 39] and MMP2 [40, 41]. Therefore, we tested whether alteration of *HOXA9* expression can affect these potential myopia markers. In the mouse RPE cells transfected with *HOXA9* cDNA, TGF-β3 was significantly increased, followed by TGF-β2 while TGF-β1 level was not affected (Fig. 4a and b, data on TGF-β1 are not shown). *FGF2* and *IGF1R* expression levels were also dose-dependently increased by *HOXA9*. *MMP2* that is consistently shown to be elevated in myopic animal models was substantially up-regulated by *HOXA9*. However, over-expression of *HOXA9* had no significant influence on *IGF1* or *HGF* expression (data not shown). Furthermore, *HOXA9* increased the RPE proliferation and viability in a dose-dependent manner (Fig. 4c and d).

## Discussion

*HOXA9* is a transcription factor and its function in the eyes has not been reported. Our data implied that *HOXA9* may play a role in myopia development. Using a FDM myopic model, we first showed that *HOXA9* expression level was significantly higher in the myopic eyes than the fellow normal eyes. Among 8 pre-school children with SE less than -2D, 7 had hypomethylation at *HOXA9* which suggested that over-expressed *HOXA9* could be a risk factor for early onset myopia. Our cellular studies further demonstrated that an increase of *HOXA9* could increase expression in several pro-myopic genes including *TGF*-β, *FGF2*, *IGF1R* and *MMP2* genes.

The *HOX* genes are a subset of homeobox genes which regulate the development of anatomical structures. There are four *HOX* gene clusters (*HOXA*, *HOXB*, *HOXC* and *HOXD*) in humans. The regulation of *HOX* genes is highly complex, which includes microRNA, DNA methylation and histone modification. The *HOXA9* gene encodes a DNA-binding transcription factor which may regulate gene expression, morphogenesis, and differentiation. The *HOXA9* gene is of particular interest from a hematopoietic perspective as its dysfunction has been implicated in acute myeloid leukemia [42]. Our data is the first to suggest that *HOXA9* may affect myopia development via multiple mechanisms including myopia-related genes and RPE proliferation. Although our findings are intriguing, more studies are needed to confirm the role of *HOXA9* in myopia development.

It has been indicated that retino-scleral signaling cascade can participate in scleral remodeling during myopic development [43]. The key location of RPE cells makes them plausible conduits for relaying growth regulatory signals originating in the retina to the sclera. In addition, RPE also represents a major source of growth factors and cytokines that can mediate the retinoscleral signaling pathway [34], including IGF1, TGF-β, FGF, and VEGF [44, 45]. We demonstrated that over-expression of *HOXA9* in RPE cells could increase pro-myopia substances including TGF-β, FGF2, IGF1R and MMP2. However, we acknowledge that our initial discovery of *HOXA9* in relation to myopia development was based on small samples of human subjects and animals. Although we show molecular evidence to support *HOXA9* as a novel myopia gene, it is warranted that future studies use more animal and human data to validate the role of *HOXA9* in myopia.

## Conclusion

To sum up, the present study showed that *HOXA9* might be a novel gene that promotes myopia development. Since *HOXA9* is a transcription factor, it may directly or indirectly affect expression of pro-myopia genes. In addition, *HOXA9* also increases cell proliferation, which may facilitate eyeball elongation during myopia development. However, further studies are warranted to validate our findings.

## Abbreviations

ARPE-19: Human retinal pigment epithelial cell line
AXL: Axial length
FDM: Form deprivation myopia
miR-328: microRNA-328
SE: Spherical equivalent refraction
TGF-β: Transforming growth factor-β
